# Development and Validation of Nomograms Based on Gamma-Glutamyl Transpeptidase to Platelet Ratio for Hepatocellular Carcinoma Patients Reveal Novel Prognostic Value and the Ratio Is Negatively Correlated With P38MAPK Expression

**DOI:** 10.3389/fonc.2020.548744

**Published:** 2020-12-03

**Authors:** Dingan Luo, Haoran Li, Jie Hu, Mao Zhang, Shun Zhang, Liqun Wu, Bing Han

**Affiliations:** ^1^Department of Hepatobiliary and Pancreatic Surgery, The Affiliated Hospital of Qingdao University, Qingdao, China; ^2^Department of General Surgery, The Third Xiangya Hospital of Central South University, Changsha, China

**Keywords:** gamma-glutamyl transpeptidase, platelet, p38 mitogen-activated protein kinase, hepatocellular carcinoma, prognosis, dynamic nomogram

## Abstract

**Background:**

Early prediction of recurrence and death risks is significant to the treatment of hepatocellular carcinoma (HCC) patients. We aimed to develop and validate prognosis nomogram models based on the gamma-glutamyl transpeptidase (GGT)-to-platelet (PLT) ratio (GPR) for HCC and to explore the relationship between the GPR and inflammation-related signaling pathways.

**Methods:**

All data were obtained from 2000 to 2012 in the Affiliated Hospital of Qingdao University. In the training cohort, factors included in the nomograms were determined by univariate and multivariate analyses. In the training and validation cohorts, the concordance index (C-index) and calibration curves were used to assess predictive accuracy, and receiver operating characteristic curves were used to assess discriminative ability. Clinical utility was evaluated using decision curve analysis. Moreover, improvement of the predictive accuracy of the nomograms was evaluated by calculating the decision curve analysis, the integrated discrimination improvement, and the net reclassification improvement. Finally, the relationship between the GPR and inflammation-related signaling pathways was evaluated using the independent-samples t-test.

**Results:**

A larger tumor size and higher GPR were common independent risk factors for both disease-free survival (DFS) and overall survival (OS) in HCC (P < 0.05). Good agreement between our nomogram models’ predictions and actual observations was detected by the C-index and calibration curves. Our nomogram models showed significantly better performance in predicting the HCC prognosis compared to other models (P < 0.05). Online webserver and scoring system tables were built based on the proposed nomogram for convenient clinical use. Notably, including the GPR greatly improved the predictive ability of our nomogram models (P < 0.05). In the validation cohort, p38 mitogen-activated protein kinase (P38MAPK) expression was significantly negatively correlated with the GPR (P < 0.01) and GGT (P = 0.039), but was not correlated with PLT levels (P = 0.063). And we found that P38MAPK can regulate the expression of GGT by quantitative real-time PCR and Western blotting experiments.

**Conclusions:**

The dynamic nomogram based on the GPR provides accurate and effective prognostic predictions for HCC, and P38MAPK-GGT may be a suitable therapeutic target to improve the prognosis of HCC patients.

## Introduction

Hepatocellular carcinoma (HCC) is one of the most common fatal cancers in the world. In China, HCC has the 4^th^ highest cancer incidence rate and the 3^rd^ highest mortality rate (1). In recent years, although significant progress has been achieved in the diagnosis and treatment of HCC, the prognosis of HCC patients is poor because of the high recurrence rate (2). Thus, early prediction and early intervention are essential for prolonging HCC patients’ long‐term survival.

In the past several years, more and more studies have focused on the gamma-glutamyl transpeptidase (GGT)-to-platelet (PLT) ratio (GPR), an inflammatory indicator, for early prediction of the prognosis of liver diseases. For example, several studies have shown that the GPR can predict cirrhosis and fibrosis in patients with hepatitis B virus infection (3, 4) and is also an independent risk factor for a poor prognosis after hepatectomy for hepatitis B virus (HBV)-related HCC (5). In fact, compared with a single factor, the combination of several independent factors can substantially improve predictive ability (6). Although some nomogram models have been formulated to predict the prognosis of HCC patients in the past several years (7, 8), unfortunately, no universal and widely recognized applicable nomogram model based on the GPR is available for this purpose. More importantly, the performance of these nomogram models is unsatisfactory. Therefore, we aimed to develop and validate excellent and effective nomogram models based on the GPR for early, personalized prediction of the prognosis of HCC patients and to identify potential therapeutic targets to improve patient survival.

Inflammation-related signaling pathways are known to be closely related to the occurrence and development of HCC (9–11), but the relationship between the GPR and inflammation-related signaling pathways remains unknown. Therefore, we also detected the expression of key molecules in inflammation-related pathways in HCC patients, such as p38 mitogen-activated protein kinase (P38MAPK), janus kinase 2 (JAK2), signal transducer and activator of transcription 3 (STAT3), nuclear factor kappa-light-chain-enhancer of activated B cells (NFκB), and inhibitor kappa B kinase β (IKKβ). Subsequently, we further studied possible correlations between the GPR and these key molecules to identify possible therapeutic targets to greatly improve the prognosis of HCC patients.

## Materials and Methods

### Patients

We reviewed HCC patients who had undergone R0 resection between January 2000 and December 2010 at the Affiliated Hospital of Qingdao University. This retrospective study was performed in line with the Helsinki Declaration and with approval from the Ethics Committee at the Affiliated Hospital of Qingdao University. All patients signed a written informed consent form before surgery. The inclusion criteria were as follows: 1) R0 resection; and 2) HCC diagnosed according to the European Society of Liver Research radiological standards and postoperative pathology; and 3) Survival more than 30 days. The exclusion criteria were as follows: 1) any anti-cancer treatment before surgery; 2) non-tumor-related death; and 3) loss to follow-up. Ultimately, 513 HCC patients were included in the training cohort. The clinicopathological information of the HCC patients, including gender, age, alpha fetoprotein (AFP) level, tumor size, and tumor number, were retrospectively included from electronic medical records. To increase the credibility of our study, we also collected a validation cohort consisting of 83 HCC patients selected according to the same inclusion and exclusion criterion above who were treated between January 2011 and December 2012. More importantly, these patients had paraffin-embedded specimens.

We calculated the GPR based on previously described methods (12): GPR = GGT/ULN of GGT/platelet count × 100. GGT is expressed in units of U/L, ULN is the upper limit of normal for that laboratory result, and PLT is expressed in units of 10 (9)/L.

### Immunohistochemistry and Scoring Criteria

Briefly, paraffin-embedded specimens from 83 HCC patients were cut into 4-μm-thick sections, dewaxed, and hydrated. After antigen retrieval in citrate buffer (10 mM, pH 6), endogenous peroxidase activity was blocked with 3% hydrogen peroxide for 10 min. Subsequently, the paraffin sections were stained overnight at 4°C with primary antibodies. The primary antibodies for P38MAPK (catalogue no. Ab197348), JAK2 (catalogue no. Ab39636), STAT3 (catalogue no. Ab119352), and NFκB (catalogue no. Ab209795) were purchased from Abcam, USA. The primary antibody for IKKβ (catalogue no. 07-1479) was purchased from Sigma-Aldrich, USA. The MaxVision kit (Fuzhou Maixin Biotechnology Development Co., Ltd., Fujian, China) was used to detect the primary antibodies, and the color was developed using 3, 3’−diaminobenzidine chromogen substrate for 10 min at room temperature. Next, the specimens were counterstained with hematoxylin for 1 min. The sections were dehydrated in graded ethanol, cleared, and mounted.

Two pathologists who were blinded to the patients’ clinical data performed simultaneous reviews and discussed the staining results. When their findings differed, a third person evaluated the samples. Ten optical fields were observed under a high-power lens (×400), and the degree of staining of positive cells and the percentage of positive cells were calculated. Cells without coloration or with a pale yellow, brownish-yellow, or brown color were assigned scores of 0, 1, 2, or 3, respectively; cells with <5, 5–25, 26–50, 51–74, or ≥75% positivity were scored as 0, 1, 2, 3, or 4, respectively. The two scores were added together, and a score ≤4 points was considered low expression, while a score >4 points was considered high expression.

### Patient Follow-Up

A close follow-up of all patients meeting the research criteria was conducted; follow-up forms were completed by outpatient consultation, telephone calls, and letters. The typical follow-up plan was as follows: a review was performed every 3 months for 2 years after surgery, every 6 months between 2 years and 5 years after surgery, and annually after 5 years postoperatively. The review included analysis of parameters such as liver function and AFP levels, abdominal ultrasounds, and chest radiographs. If necessary, enhanced computed tomography (CT), magnetic resonance imaging (MRI), needle biopsy, or other procedures were ordered. Imaging or biopsy was performed to identify recurrence and to confirm new lesions inside or outside of the liver. The disease-free survival (DFS) was defined as the time from the operation to recurrence or the time from the operation to the end of follow-up. The overall survival (OS) was defined as the time from the operation to death or the time from the operation to the end of follow-up. DFS and OS were calculated on a monthly basis, and the follow-up endpoint was April 2017.

### Cell Culture and Reagents

Liver cancer cell lines (HepG2) were purchased from a cell bank at the Chinese Academy of Sciences (Shanghai, China). HepG2 was cultured in Minimum Essential Medium with 10% FBS and 1% P/S and deposed in a humidified atmosphere with 5% CO2 at 37°C.

### Transfection

The p38-siRNA and control siRNA were acquired from Shanghai Gene Pharma Company (China). Transfection was performed according to the manufacturer’s instructions for the use of the Lipofectamine 2000 (Invitrogen, Carlsbad, CA, USA). Cells were transfected with p38-siRNA for 24 h and harvested for subsequent experiments. The p38-siRNA sequences were as follows: GGUCAGUGGGAUGCAUAAUTT/AUUAUGCAUCCCACUGACCTT. The control siRNA sequences were as follows: UUCUCCGAACGUGUCACGUTT/ACGUGACACGUUCGGAGAATT.

### RNA Extraction and Quantitative Real-Time PCR (qPCR)

Total RNA from cultured cells and frozen tissues was extracted with TRIzol (Invitrogen, Carlsbad, CA, USA). cDNA synthesis was performed using the PrimeScript™ RT Kit (TaKaRa, Otsu, Japan). SYBR Premix EX Taq™ (TaKaRa, Otsu, Japan) was used for qPCR on an FTC-3000p RealTime PCR system (Funglyn Biotech, Shanghai, China). Relative gene expression was determined by the comparative 2^−ΔΔCT^ method. The primer sequences were as follows: p38MAPK-F: CGGAGAGGTTCCATATTGGGT; p38MAPK-R: TTCCAAGAGCCAGCAAACGG; GGT1-F: AGCCCAGAAGTGAGAGCAGTTG; GGT1-R: ACCTGAGCTTCCCCACCTATGA; GAPDH-F: TGACTTCAACAGCGACACCCA; GAPDH-R: CACCCTGTTGCTGTAGCCAAA.

### Western Blotting Analysis

Western blotting analysis of protein expression was performed as described previously (13). Briefly, protein lysates were separated using sodium dodecyl sulfate-polyacrylamide gel electrophoresis, and target proteins were detected by western blotting with antibodies against p38MAPK (Abcam, Ab197348), p-p38MAPK (Cell Signaling Technology, 4511), GGT (KleanAB, P100867), and β-actin (Sigma-Aldrich, A3854).

### Statistical Analysis

**Figure 1:**
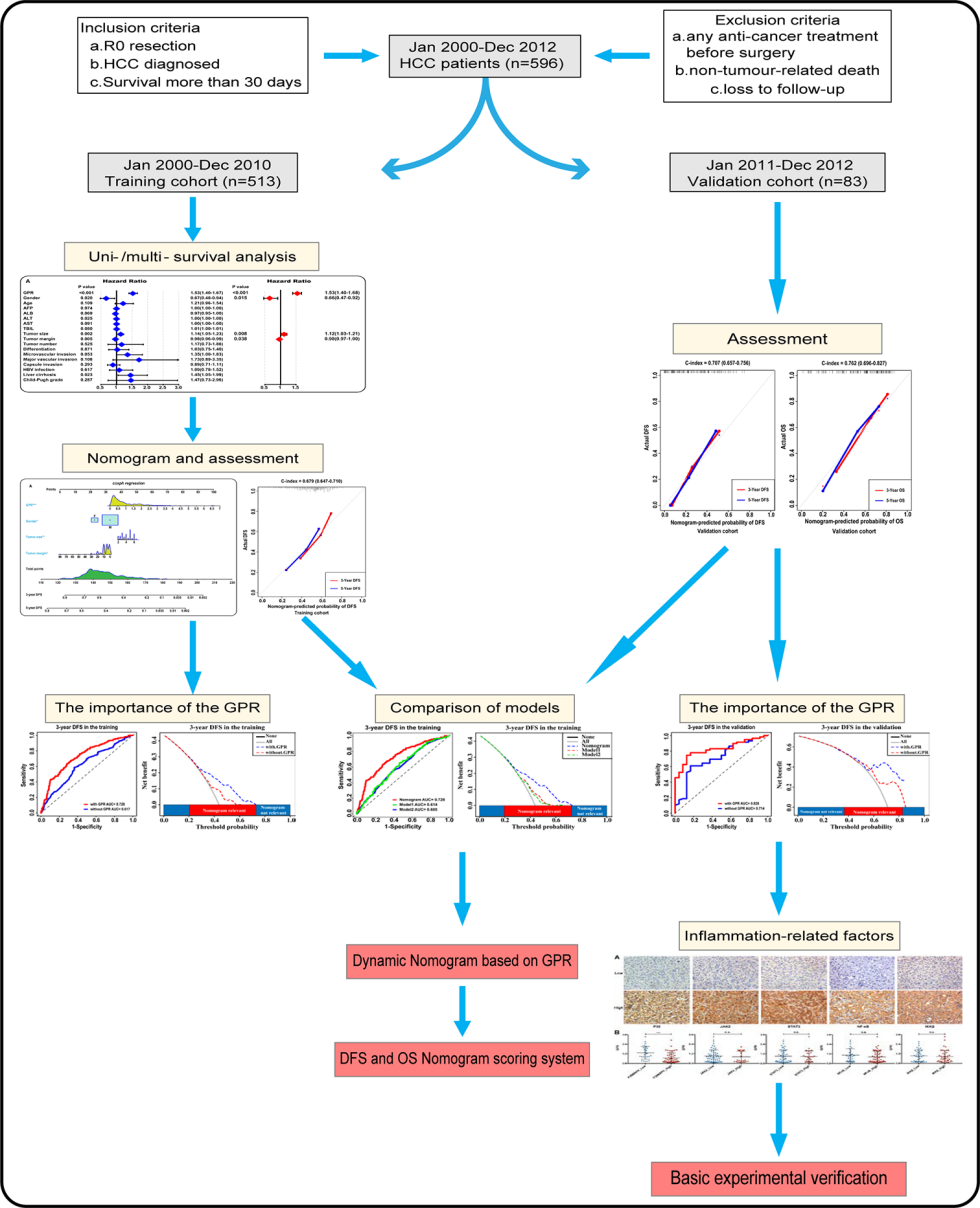
Flowchart of the analysis. GPR, gamma‐glutamyl transpeptidase‐to‐platelet ratio; HCC, hepatocellular carcinoma.

Statistical analysis was performed using SPSS (Version 24.0, IBM Co. NY, USA) and R (Version 3.5.1, R Development Core Team), and P < 0.05 (two-tailed) was considered to denote a significant difference. Continuous variables were presented as the mean ± SD and tested by Student’s t-test. Categorical variables were expressed as a number (%) and analyzed by Fisher’s exact test or the χ2 test. Univariate and multivariate stepwise regression analysis were used to determine the significant factors associated with prognosis. Based on the results of the multivariate analysis, two nomograms were constructed to predict 3- and 5-year DFS and OS rates after hepatectomy. Internal validation was evaluated by the C-index and calibration curves. Bootstrap resampling (1,000 resamples) was used for the calibration curves. The discriminatory capabilities of the nomograms and other variables were assessed with receiver operating characteristic (ROC) curves, and the clinical utility of the nomograms and other variables was carefully investigated using decision curve analysis (DCA) to compensate for the limitations of ROC curves. Moreover, improvement of the predictive accuracy of the nomograms was evaluated by calculating the DCA, the integrated discrimination improvement (IDI), and the net reclassification improvement (NRI). Importantly, the results were further confirmed in the validation cohort. Work of flow is displayed in **Figure 1**.

## Results

### Patient Characteristics

In our study, the mean age of all 596 HCC patients (513 in the training cohort and 83 in the validation cohort) was 55.0 ± 10.1 years, 84.9% (506/596) of the patients were male, and 87.8% of the patients (523/596) were positive for HBV surface antigen. The mean follow‐up was 69.4 ± 41.6 months for all patients. The 3‐ and 5‐year DFS rates were 51.0% (304/596) and 38.1% (227/596), respectively, and the 3‐ and 5‐year OS rates were 75.3% (449/596) and 61.2% (365/596), respectively. The baseline characteristics of all HCC patients are listed in **Table 1**, which did not significantly differ between the training and validation cohorts.

**Table 1:** Basic characteristics of HCC patients in the training and validation cohort.

Variables	Training cohort (n = 513)	Validation cohort (n = 83)	T/χ^2^ Test	*P* value
Gender			0.236	0.628
Male	437 (85.2%)	69 (83.1%)		
Female	76 (14.8%)	14 (16.9%)		
Age (years)	55.1 ± 10.2	54.7 ± 9.7	0.297	0.767
AFP (ng/L)	242.1 ± 408.5	298.8 ± 466.1	-1.047	0.298
ALB (g/L)	39.4 ± 3.8	38.7 ± 3.8	1.542	0.124
ALT (U/L)	50.9 ± 43.2	50.4 ± 34.9	0.109	0.913
AST (U/L)	42.2 ± 42.8	39.9 ± 29.8	0.462	0.644
GGT (U/L)	68.1 ± 72.3	56.8 ± 63.1	1.348	0.178
PLT (10^9^/L)	146.3 ± 71.3	135.1 ± 92.9	1.052	0.295
TBIL (μmol/L)	17.4 ± 17.9	16.6 ± 6.2	0.410	0.682
Tumor size (cm)	4.1 ± 1.3	3.8 ± 1.6	1.633	0.103
Tumor margin (mm)	7.5 ± 7.7	8.8 ± 8.6	−1.397	0.163
Tumor number			0.000	1.000
Single	493 (96.1%)	80 (96.4%)		
Multiple	20 (3.9%)	3 (3.6%)		
Differentiation			0.842	0.359
High and middle	438 (85.4%)	74 (89.2%)		
Low	75 (14.6%)	9 (10.8%)		
Microvascular invasion			0.464	0.496
Yes	72 (14.0%)	14 (16.9%)		
No	441 (86.0%)	69 (83.1%)		
Major vascular invasion			1.136	0.287
Yes	11 (2.1%)	4 (4.8%)		
No	502 (97.9%)	79 (95.2%)		
Capsule invasion, yes/no			0.133	0.715
Yes	332 (64.7%)	52 (62.7%)		
No	181 (35.3%)	31 (37.3%)		
HBV infection			0.177	0.674
Yes	449 (87.5%)	74 (89.2%)		
No	64 (12.5%)	9 (10.8%)		
Liver cirrhosis, yes/no			0.031	0.861
Yes	59 (11.5%)	9 (10.8%)		
No	454 (88.5%)	74 (89.2%)		
Child-Pugh grade			0.000	1.000
A	500 (97.5%)	81 (97.6%)		
B	13 (2.5%)	2 (2.4%)		

HCC, hepatocellular carcinoma; AFP, α-fetoprotein; ALB, albumin; ALT, alanine aminotransferase; AST, aspartate aminotransferase; GGT, gamma-glutamyl transpeptidase; PLT, platelet; TBIL, total bilirubin; HBV, hepatitis B virus.

### Univariate and Multivariate Analysis in the Training Cohort

**Figure 2:**
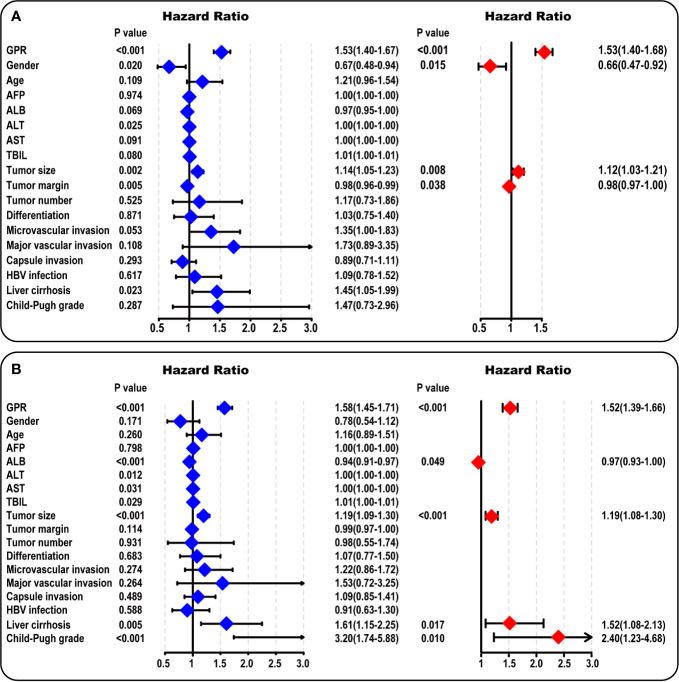
Univariate and multivariate analysis in the training cohort. **(A)** The forest map results of the univariate and multivariate survival analysis of DFS among HCC patients are shown. **(B)** The forest map results of the univariate and multivariate survival analysis of OS among HCC patients are shown. AFP, α-fetoprotein; ALB, albumin; ALT, alanine aminotransferase; AST, aspartate aminotransferase; TBIL, total bilirubin; HBV, hepatitis B virus; GPR, gamma‐glutamyl transpeptidase‐to‐platelet ratio; HCC, hepatocellular carcinoma.

According to the univariate survival analysis in the training cohort, male sex, a higher alanine aminotransferase (ALT) level, a larger tumor size, a larger tumor margin, the presence of liver cirrhosis, and a higher GPR had statistically significant effects on DFS (P < 0.05, **Figure 2A**-left). Moreover, higher albumin (ALB), ALT, aspartate aminotransferase (AST), and total bilirubin (TBIL) levels, a larger tumor size, the presence of liver cirrhosis, Child-Pugh B grade, and a higher GPR had statistically significant effects on OS (P < 0.05, **Figure 2B**-left). Subsequently, a multivariate stepwise regression analysis was performed using the variables that were statistically significant in the univariate analysis. We found that only male sex (hazard ratio (HR): 1.52, 95% CI: 1.09–2.13, P = 0.015), a larger tumor size (HR: 1.12, 95% CI: 1.03–1.21, P = 0.008), a larger tumor margin (HR: 0.98, 95% CI: 0.97–1.00, P = 0.038), and a higher GPR (HR: 1.53, 95% CI: 1.40–1.68, P < 0.001) were independent risk factors for DFS (**Figure 2A**-right). In addition, a higher ALB level (HR: 0.97, 95% CI: 0.93–1.00, P = 0.049), a larger tumor size (HR: 1.19, 95% CI: 1.08–1.30, P < 0.001), the presence of liver cirrhosis (HR: 1.52, 95% CI: 1.08–2.13, P = 0.017), Child-Pugh B grade (HR: 2.40, 95% CI: 1.23–4.68, P = 0.010), and a higher GPR (HR: 1.52, 95% CI: 1.39–1.66, P < 0.001) were independent risk factors associated with OS (**Figure 2B**-right). Interestingly, a larger tumor size and a higher GPR were common independent risk factors for both DFS and OS in HCC.

### Construction and Assessment of the Nomogram Models

**Figure 3:**
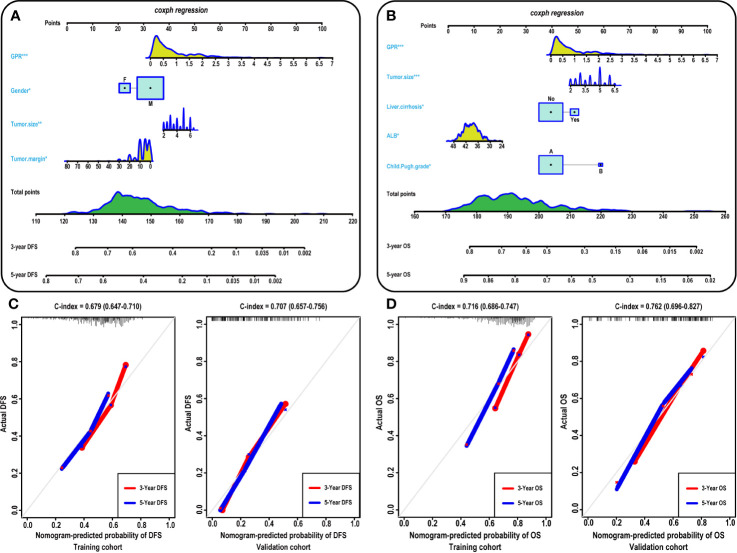
Construction and calibration of the nomogram models. **(A–B)** Development of the nomogram to predict the 3- and 5-year DFS **(A)** and OS **(B)** rates for HCC. Calibration curves showing the predictive accuracy for the 3- and 5-year DFS rates of the HCC patients in the training **(C-left)** and validation **(C-right)** cohort. Calibration curves showing the predictive accuracy for the 3- and 5-year OS rates of the HCC patients in the training **(D-left)** and validation **(D-left)** cohort. ALB, albumin; GPR, gamma‐glutamyl transpeptidase‐to‐platelet ratio; DFS, disease-free survival; OS, overall survival; HCC, hepatocellular carcinoma.

To provide a clinically relevant quantitative method to predict 3- and 5-year DFS (**Figure 3A**) and OS (**Figure 3B**) rates, two nomogram models were built according to the results of the multivariate analysis in the training cohort. Each independent risk factor got a score after nomogram calculation. After calculating the total score, we drew a vertical line according to the total score and obtained the probabilities of individual recurrence and mortality.

Furthermore, the prognostic accuracy of the models for DFS and OS was calculated using the C-index from internal validation, and the C-index values for DFS and OS were 0.679 (0.647–0.710) and 0.716 (0.686–0.747) in the training cohort, respectively, indicating that approximately 67.9 and 71.6% of the probabilities of individual recurrence and mortality would be correctly predicted by the nomogram models, respectively. In the validation cohort, the C-index values were 0.707 and 0.762 for DFS and OS, respectively, demonstrating the excellent predictive ability of our nomogram models. For the training cohort, the predictive accuracy for the 3- and 5-year DFS (**Figure 3C**-left) and OS (**Figure 3D**-left) rates is shown by the calibration curves in the internal validation. The calibration plots showed high consistency between predictions of the probability of individual HCC recurrence and mortality and the actual observed results. Moreover, in the validation cohort, the calibration curves also showed high consistency between predictions of the probability of individual HCC recurrence (**Figure 3C**-right) and mortality (**Figure 3D**-right) and the actual observed results.

### Comparison Between the Nomogram Models and Other Models

**Figure 4:**
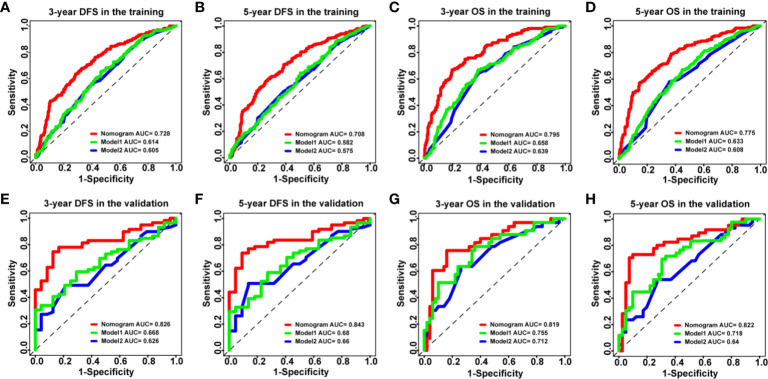
The ROC curves of the nomogram models and other models in HCC patients. **(A–D)** ROC curves showing the discriminatory abilities of the nomogram models and other models for the 3-year DFS **(A)** and OS **(C)** rates and 5-year DFS **(B)** and OS **(D)** rates in the training cohort. **(E–H)** ROC curves showing the discriminatory abilities of the nomogram models and other models for the 3-year DFS **(E)** and OS **(G)** rates and 5-year DFS **(F)** and OS **(H)** rates in the validation cohort. GPR, gamma‐glutamyl transpeptidase‐to‐platelet ratio; ALB, albumin; ROC, receiver operating characteristic; HCC, hepatocellular carcinoma; DFS, disease-free survival; OS, overall survival.

To further validate the superiority of our nomograms in assessing the prognosis of HCC patients, in the training and validation cohorts, we plotted not only the ROC curves of our nomogram model but also the ROC curves of other nomogram models (Model 1 (7) and Model 2 (8)), which have been published previously. The discriminatory ability of each model was evaluated by the Area Under Curve (AUC). Interestingly, the 3- and 5-year AUCs for our nomograms were significantly higher than those for Model 1 and Model 2 in the training and validation cohorts (**Figure 4**). The above evidence reflects the high diagnostic value of our nomogram models. We also use C-index values to further verify the advantage of our nomograms in evaluating the prognosis of HCC patients (**Table 2**).

**Table 2:** Comparison of the C-index of Nomogram, Model 1, and Model 2.

Models	Training cohort		Validation cohort	
	C-index (95% CI)	P value	C-index (95% CI)	P value
**Disease-free survival**				
Nomogram	0.679 (0.647–0.710)		0.707 (0.657–0.756)	
Model 1	0.591 (0.560–0.622)	**<0.001**	0.657 (0.595–0.720)	**0.048**
Model 2	0.586 (0.552–0.621)	**<0.001**	0.632 (0.563–0.702)	**0.013**
**Overall survival**				
Nomogram	0.716 (0.686–0.747)		0.762 (0.696–0.827)	
Model 1	0.619 (0.586–0.653)	**<0.001**	0.690 (0.614–0.766)	**0.019**
Model 2	0.595 (0.557–0.633)	**<0.001**	0.652 (0.570–0.734)	**0.004**

Bold indicates that the P value is statistically significant.

**Figure 5:**
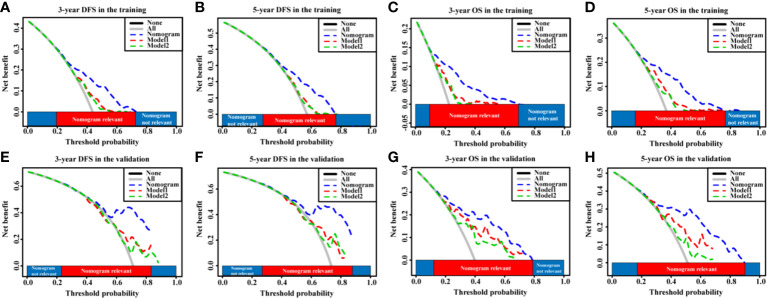
The DCA curves of the nomogram models and other models in HCC patients. **(A–D)** DCA curves showing the clinical utility of the nomogram models other models for the 3-year DFS **(A)** and OS **(C)** rates and 5-year DFS **(B)** and OS **(D)** rates in the training cohort. **(E–H)** DCA curves showing the clinical utility of the nomogram models and other models for the 3-year DFS **(E)** and OS **(G)** rates and 5-year DFS **(F)** and OS **(H)** rates in the validation cohort. GPR, gamma‐glutamyl transpeptidase‐to‐platelet ratio; ALB, albumin; DCA, decision curves analysis; HCC, hepatocellular carcinoma; DFS, disease-free survival; OS, overall survival.

In addition, according to the DCA, the nomograms showed exceptional performance in the training cohort (**Figures 5A–D**) and the validation cohort (**Figures 5E–H**) compared with models regardless of the threshold, ensuring achievement of the maximum clinical benefit. Overall, the ROC, the C-index, and the DCA curves indicated that our nomograms enabled valuable predictions.

### Development of Webserver and Scoring System for Easy Access of Our New Model

Online version of our nomogram (**Supplementary Figure 1**) can be accessed at https://00logan00.shinyapps.io/Article_GPR_DFS/ and https://00logan00.shinyapps.io/Article_GPR_OS/, to assist researchers and clinicians. Predicted the probability of DFS (**Supplementary Figure 1A**) and OS (**Supplementary Figure 1B**) across time can be easily determined by inputting clinical features and reading output figures and tables generated by the webserver.

In addition, we constructed two scoring tables to predict the probability of DFS (**Table 3**) and OS (**Table 4**). The physicians could perform an individualized survival prediction through this easy-to-use scoring system.

**Table 3:** DFS nomogram scoring system.

Gender	Points	Tumor size	Points	Tumor margin	Points	GPR	Points
Female	23	2	38	0	33	0	33
Man	33	4	43	20	24	1	43
		6	48	40	16	2	53
		7	51	60	8	3	63
				80	0	4	74
						5	84
						6	94
						7	104
**Total points**		**3-year DFS**		**Total points**		**5-year DFS**	
110		0.88		110		0.825	
120		0.826		120		0.747	
130		0.748		130		0.642	
140		0.644		140		0.510	
150		0.512		150		0.359	
160		0.362		160		0.212	
170		0.214		170		0.095	
180		0.096		180		0.028	
190		0.029		190		0.004	
200		0.005		200		<0.001	
210		<0.001		210		<0.001	
220		<0.001		220		<0.001	

DFS predictions corresponding to total points not shown in the table may be obtained by linear interpolation. The same approach may be followed for obtaining specific points for values of age not included here. Abbreviations: GPR, gamma-glutamyl transpeptidase-to-platelet ratio; DFS, Disease-free survival.

Table 4OS nomogram scoring system.

**Table:** 

Liver cirrhosis	Points	Child-Pugh	Points	Tumor size	Points	ALB	Points	GPR	Points
No	38	A	38	2	46	24	20	0	38
Yes	48	B	58	5	57	36	11	1	48
				7	65	48	2	2	57
								3	66
								4	76
								5	85
								6	94
								7	104

**Table:** 

Total points	3-year OS	Total points	5-year OS
150	0.957	150	0.919
160	0.934	160	0.877
170	0.898	170	0.814
180	0.846	180	0.725
190	0.769	190	0.604
200	0.663	200	0.454
210	0.526	210	0.291
220	0.365	220	0.145
230	0.207	230	0.049
240	0.085	240	0.009
250	0.021	250	0.001
260	0.002	260	<0.001

OS predictions corresponding to total points not shown in the table may be obtained by linear interpolation. The same approach may be followed for obtaining specific points for values of age not included here. Abbreviations: ALB, albumin; GPR, gamma-glutamyl transpeptidase-to-platelet ratio; OS, Overall survival.

### The Importance of the GPR to Enhance the Predictive Performance of Models

**Figure 6:**
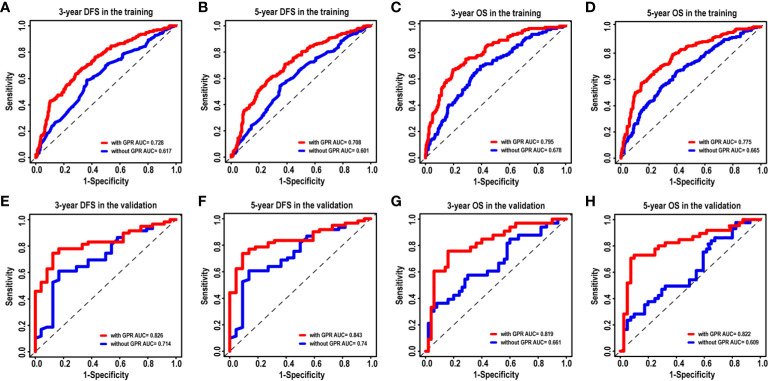
The ROC curves of the nomogram models with and without the GPR for HCC patients. **(A–D)** ROC curves showing the discriminatory abilities of the nomogram models with and without the GPR for the 3-year DFS **(A)** and OS **(C)** rates and 5-year DFS **(B)** and OS **(D)** rates in the training cohort. **(E–H)** ROC curves showing the discriminatory abilities of the nomogram model with and without the GPR for the 3-year DFS **(E)** and OS **(G)** rates and 5-year DFS **(F)** and OS **(H)** rates in the validation cohort. GPR, gamma‐glutamyl transpeptidase‐to‐platelet ratio; ROC, receiver operating characteristic; HCC, hepatocellular carcinoma; DFS, disease-free survival; OS, overall survival.

**Figure 7:**
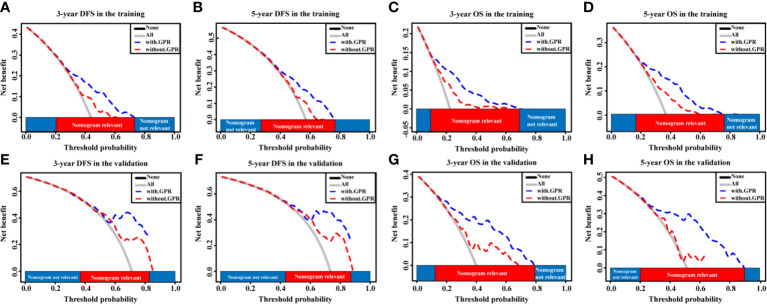
The DCA curves of the nomogram models with and without the GPR for HCC patients. **(A–D)** DCA curves showing the clinical utility of the nomogram models with and without the GPR for the 3-year DFS **(A)** and OS **(C)** rates and 5-year DFS **(B)** and OS **(D)** rates in the training cohort. **(E–H)** DCA curves showing the clinical utility of the nomogram models with and without the GPR for the 3-year DFS **(E)** and OS **(G)** rates and 5-year DFS **(F)** and OS **(H)** rates in the validation cohort. GPR, gamma‐glutamyl transpeptidase‐to‐platelet ratio; ALB, albumin; DCA, decision curves analysis; HCC, hepatocellular carcinoma; DFS, disease-free survival; OS, overall survival.

Given that our nomograms perform better than other models in predicting the prognosis of HCC, we further verified whether inclusion of the GPR can greatly affect the predictive ability of our nomogram models. Therefore, we plotted the ROC curves of the nomogram models with and without the GPR in the training cohort (**Figures 6A–D**). The AUCs for the nomogram with the GPR in the training cohort (3-year DFS: AUC = 0.728, **Figure 6A**; 5-year DFS: AUC = 0.708, **Figure 6B**; 3-year OS: AUC = 0.795, **Figure 6C**; 5-year OS: AUC = 0.775, **Figure 6D**) was all significantly higher than those for the nomogram without the GPR. In addition, when the NRI and IDI were analyzed, which are more sensitive than the other methods used in this study, we found that including the GPR can significantly improve the predictive accuracy of the nomogram models (**Table 5**). Compared with the nomogram without the GPR in the training cohort, the nomogram with the GPR yielded an IDI of 6% and an NRI of 20% (P < 0.001, 3-years DFS) or an IDI of 5% and an NRI of 14% (P < 0.001, 3-years OS); the nomogram with the GPR yielded an IDI of 6% and an NRI of 19% (P < 0.001, 5-years DFS) or an IDI of 7% and an NRI of 30% (P < 0.001, 5-years OS). We also used the DCA to assess the potential clinical effects of the nomograms with or without the GPR in the training cohort, and our results showed that the nomograms with GPR showed exceptional performance (**Figures 7A–D**). Just as importantly, we got the same result in the validation cohort (**Figures 6E–H**, **Figures 7E–H**). Our results indicated that the GPR had an indispensable role in these models by substantially enhancing their predictive performance.

**Table 5:** Comparison of the NRI and IDI of the nomogram model with and without GPR.

With GPR *vs.* Without GPR	Training cohort				Validation cohort			
	NRI	*P* value	IDI	*P* value	NRI	*P* value	IDI	*P* value
**Disease-free survival**								
3-years	0.20	<0.001	0.06	**<0.001**	0.53	<0.001	0.16	**<0.001**
5-years	0.19	<0.001	0.06	**<0.001**	0.31	0.016	0.17	**<0.001**
**Overall survival**								
3-years	0.14	<0.001	0.05	**<0.001**	0.66	<0.001	0.18	**<0.001**
5-years	0.30	<0.001	0.07	**<0.001**	0.61	<0.001	0.20	**<0.001**

IDI, integrated discrimination improvement; NRI, net reclassification improvement; GPR, gamma-glutamyl transpeptidase to platelet ratio.

Bold indicates that the P value is statistically significant.

### The Relationship Between the GPR and Inflammation-Related signaling pathways

**Figure 8:**
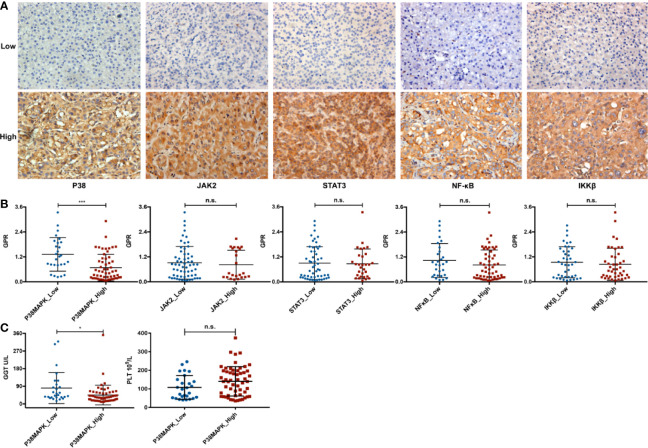
The relationship between the GPR and inflammation-related signaling pathways. **(A)** representative immunohistochemistry images of the P38MAPK, JAK2, STAT3, NFκB, and IKKβ proteins in HCC tissue specimens (400×). **(B)** the comparison between the above five key inflammation-related factors and the GPR. **(C)** the comparison between P38MAPK expression and GGT or PLT levels. P38MAPK, p38 mitogen-activated protein kinase; JAK2, janus kinase 2; STAT3, signal transducer and activator of transcription 3; NFκB, nuclear factor kappa-light-chain-enhancer of activated B cells; IKKβ, inhibitor kappa B kinase β; GPR, gamma‐glutamyl transpeptidase‐to‐platelet ratio; GGT, gamma-glutamyl transpeptidase; PLT, platelet; HCC, hepatocellular carcinoma.

To evaluate the relationship between the GPR and inflammation-related signaling pathways, the HCC patients in the validation cohort were divided into low-expression and high-expression subgroups according to the expression levels of key molecules (**Figure 8A**). The mean GPRs of the low- and high-expression groups were compared using the independent-samples t-test. Notably, a high mean GPR of 1.32 ± 0.81 was found in the low P38MAPK expression group, while a low mean GPR of 0.68 ± 0.64 was found in the high P38MAPK expression group, and the difference was statistically significant (P = 0.000, **Figure 8B**), revealing a negative correlation between the GPR and P38MAPK expression. However, no correlation was found between the GPR and the expression of key factors in other signaling pathways, such as JAK2, STAT3, NFκB, or IKKβ (P > 0.05, **Figure 8B**). Interestingly, the GPR comprises GGT and PLT levels, and P38MAPK expression was significantly negatively correlated with GGT levels (P = 0.039, **Figure 8C**), but was not correlated with PLT levels (P = 0.063, **Figure 8C**).

In addition, we further divided the high and low groups according to the average value of GPR, and conducted chi-square test with P38MAPK. We found that there is also a correlation between GPR and P38 (**Supplementary Table 1**).

### Verification of the Relationship Between GGT and P38MAPK

**Figure 9:**
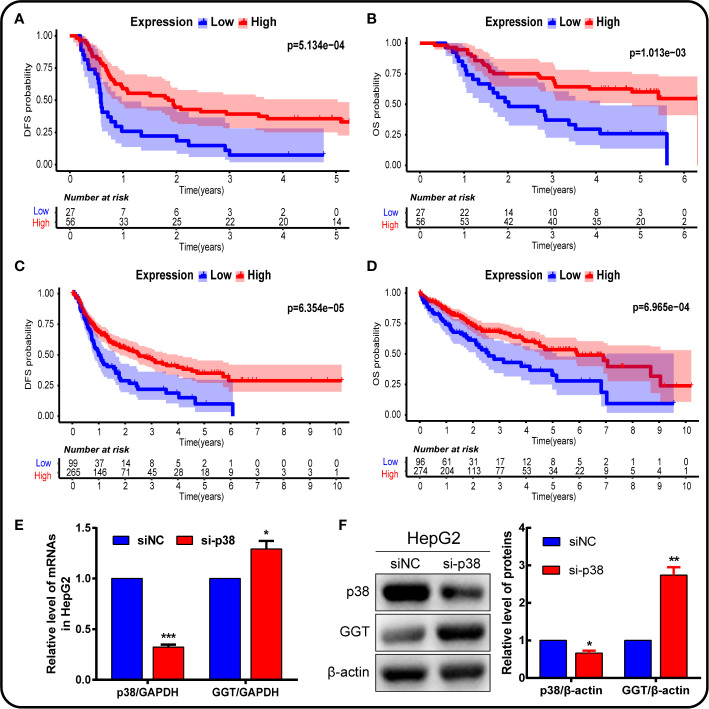
P38MAPK plays a vital role in prognosis and regulates GGT expression in HepG2 hepatoma cells. The prognosis of P38MAPK expression was analyzed using own data **(A, B)** and data from TCGA database **(C, D)**. **(E)** qPCR analysis of p38MAPK and GGT mRNA expression in HepG2 cells transfected with si-p38MAPK and siNC for 24 h. GAPDH was used as a loading control. **(F)** Western blotting analysis of p38MAPK and GGT protein expression in HepG2 cells transfected with si-p38MAPK and siNC for 24 h. β-Actin was used as a loading control.

We further analyzed the impact of P38MAPK on the prognosis using IHC data (**Figures 9A, B**), and verified the data using the TCGA mRNA data (**Figures 9C, D**). The higher the expression of P38MAPK, the lower the patient’s prognosis risk. We found that P38MAPK can be a potential therapeutic target to improve the prognostic survival rate of patients.

**Figure 10:**
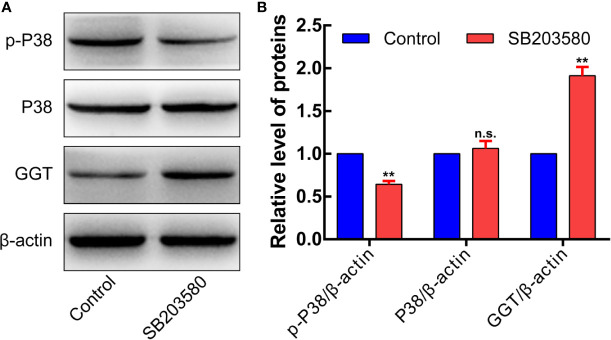
p-P38MAPK is a regulator of the GGT. To further understand how P38 regulates GGT, we treated cells with P38 inhibitors, SB203580 (20 μM), for 24 h. Western blot analysis results showed that the protein levels of p-p38 decreased significantly, while GGT increased after treatment with SB203580 **(Panels 10A, B)**.

In addition, cells were transfected with siRNA specifically targeting P38 to knock down the P38 expression. Following P38 knockdown with si-P38 for 24 h, the expression levels of GGT were markedly increased in HepG2 cells (**Figures 9E, F**). To further understand how P38 regulates GGT, we treated cells with P38 inhibitors, SB203580 (20 μM), for 24 h. Western blot analysis results showed that the protein levels of p-p38 decreased significantly, while GGT increased after treatment with SB203580 (**Figures 10A, B**). These data suggest that P38 is a regulator of the GGT.

## Discussion

Accurate prognostic assessment is essential for selecting appropriate treatment strategies for HCC patients. In this study, we comprehensively evaluated the relationship between some routine and easily available clinicopathological indicators and the prognosis of HCC. A nomogram model based on GPR was developed, which provides accurate and effective prognostic prediction for HCC. Compared with other models, it shows significantly better performance. It’s worth noting that we have developed a webserver and scoring system to allow easy access to our new model. Interestingly, we also evaluated the expression of key factors in inflammation-related signaling pathways in the validation cohort and found that the P38MAPK inflammation-related pathway was significantly negatively correlated with GPR (P < 0.01) and GGT (P = 0.039). This suggests that P38MAPK may be a suitable therapeutic target to improve the prognosis of HCC patients.

Existing evidence suggests that a nomogram is simple, intuitive, and understandable for clinicians, and can effectively predict the patient’s prognosis (14). To our knowledge, the present study is the first to develop nomograms based on the GPR to predict the prognosis of HCC. In the multivariable regression model, we observed that male sex, a larger tumor size, a larger tumor margin, and a higher GPR were independent risk factors for DFS, and that a higher ALB level, a larger tumor size, the presence of liver cirrhosis, Child-Pugh B grade, and a higher GPR were independent risk factors for OS. Unexpectedly, we found that a larger tumor size and a higher GPR were common independent risk factors for both DFS and OS in HCC. The impact of tumor size on prognosis remains controversial. Some studies have reported that for solitary HCCs, an increasing tumor size did not decrease survival after surgery (15, 16). Furthermore, the BCLC staging system classified single tumors into stage A HCC regardless of tumor size (17). However, another study reported a contrasting opinion and suggested that large solitary tumors (>5 cm) should be classified as BCLC stage B HCC (18). Moreover, a previous study found that tumors exceeding various size thresholds, such as 8 cm, had negative impacts on the HCC prognosis (19). Unlike conventional studies, tumor size was a continuous variable in our study. Importantly, our results showed that a larger tumor size was an independent risk factor associated with prognosis. In other words, a larger tumor corresponds to a worse prognosis. The GPR is an emerging parameter that reflects systemic inflammation in HCC patients. A few studies (5, 20) have evaluated the predictive value of the GPR in patients with HBV-associated HCC by dividing the patients into low-risk and high-risk groups according to a GPR cut-off value. The results indicated that the relative risk of HCC development in the high-risk GPR group was significantly higher than in the low-risk GPR group, and these observations are consistent with our results. We conducted a long-term follow-up of our patients lasting more than 5 years and found that the GPR (continuous variable) was a major risk factor for a poor prognosis for HCC patients.

Subsequently, when all independent risk variables discussed above were incorporated into the nomogram models, we achieved C-index values of 0.679 for DFS and 0.716 for OS in the training cohort. Satisfactory verification with good calibration (C-index for DFS, 0.707; C-index for OS, 0.762) was also observed in the independent validation cohort. Moreover, in both the training cohort and the validation cohort, the AUCs shown that the nomogram models exhibited substantially better performance in prediction of the HCC prognosis compared to other models. In the present study, we also used DCA curves to evaluate potential clinical effects of the nomogram and obtained similar results. More importantly, by comparing the AUC, NRI, IDI values, and the DCA of the models with and without the GPR, we found that the predictive ability can be significantly improved by including the GPR in the models. Therefore, our findings showed that our nomogram models were more useful than other models in predicting the prognosis of HCC, and that inclusion of the GPR can greatly improve the predictive ability.

Additionally, we also examined the relationships between the GPR and common inflammation-related signaling pathways in the validation cohort, including the MAPK, JAK-STAT, NFκB, and IKKβ pathways. Interestingly, a highly significant negative correlation was found between the GPR and P38MAPK expression, and the GGT component of the GPR was found to be significantly negatively correlated with P38MAPK expression. Notably, the MAPK pathway is an important signal transduction pathway in cells primarily consisting of P38MAPK, ERK, and JNK (21), and P38MAPK signaling is an important component of the MAPK cascade, which mediates diverse biological functions (22). P38MAPK and ERK are closely related to cell invasion, migration, and apoptosis in HCC (9–11). TNF-α activates MAPKKK upstream of P38MAPK, ultimately activating P38MAPK and inducing apoptosis (23).

We further analyzed the impact of P38MAPK on the prognosis using our own data, and verified the data using the TCGA database. The higher the expression of P38MAPK, the lower the patient’s prognosis risk. The P38MAPK can be a potential therapeutic target to improve the prognostic survival rate of patients. This is also consistent with the research conclusions of other scholars (24). But some researchers found that Increased levels of p38 have been correlated with malignancy in various cancers (25, 26). The above information illustrates how the tumor suppressive mechanisms of normal cells can sometimes be switched to promote survival in cancer cells. Why p38 pathway activation induces apoptosis in some cases, but can lead to increased survival in others, is likely to depend on cell context, tumor cell type, and tumor stage (27). GGT is mainly found in the liver and is essential for maintaining cysteine levels in the body. GGT is usually expressed on the upper surface of ducts and glands, which preserves the amino acids in glutathione in duct fluid. GGT enables tumor to obtain more cysteine and cysteine from blood and interstitial fluid, and the expression of GGT provides tumor cells with selective advantages (28). Similarly, increased GGT expression is related to tumor cell resistance to cytotoxic drugs (29). The consistent research is the higher the expression of GGT, the higher the patient’s prognosis risk (30). GGT is also associated with the formation and progression of liver cancer and therefore serves as an important marker of HCC (28, 31, 32). In addition, there are reports that P38MAPK is located upstream of GGT and regulates GGT expression (33, 34). But our research found that GGT will increase with p38MAPK knockdown. It is likely to depend on cell type-specific differences, together with the intensity and duration of the signal and its crosstalk with other signaling pathways (27). We hypothesized that the possible reason for the negative correlation between the expression of GPR and P38MAPK is that the activation of the P38MAPK pathway down-regulates the expression of GGT, which in turn reduces the resistance of tumor cells and promotes HCC cell apoptosis. However, this speculation remains to be confirmed by large-scale clinical data and related experimental studies.

Although our nomograms showed good performance for HCC prognosis prediction, some limitations still should be noted in our study. First, our data were retrospectively collected from HCC patients at a single center, which may cause a selection bias. Therefore, these nomograms should be further investigated in multicenter and prospective settings in the future. Second, our data set only includes patients who underwent surgical resection for HCC; thus, this cohort of patients cannot represent HCC patients with nonresectable tumors or those who refused surgical intervention for various reasons. Third, the mechanisms underlying the associations of the GPR with P38MAPK expression and tumor progression were not clearly elucidated in our study, and further investigations may provide more information for a better understanding of the roles of GPR and P38MAPK in the development and progression of HCC.

In summary, the GPR was independent risk indicator for 3- and 5‐year DFS and OS in HCC patients. This study is the first to develop and validate excellent and effective nomogram models based on the GPR for early prediction of the prognosis risk of HCC patients. And we have developed a webserver and scoring system to easily access our new model. The models may be very beneficial for promoting precise prevention and personalized treatment strategies for HCC. Moreover, based on the negative correlation between the GPR and P38MAPK expression, P38MAPK may be a suitable therapeutic target to improve the prognosis of HCC patients.

## Data Availability Statement

The raw data supporting the conclusions of this article will be made available by the authors, without undue reservation.

## Ethics Statement

The studies involving human participants were reviewed and approved by the Ethics Committee of The Affiliated Hospital of Qingdao University. The patients/participants provided their written informed consent to participate in this study.

## Author Contributions

All authors helped perform the research. BH, LW, and SZ contributed to the study design. DL, MZ, HL, and SZ contributed to the data analysis. HL and JH contributed to the collection of tissue samples and patient data. DL and MZ wrote the manuscript, and edited the manuscript. All authors contributed to the article and approved the submitted version.

## Funding

This work was supported by the Science and Technology for People’s Livelihood Project of Qingdao under grant no. 18-6-1-89-nsh, the Key Research and Development Plan of Shandong Province under grant no. 2018GSF118233, and the Science and Technology Plan of Qingdao City Shinan District under grant no. 20184018YY.

## Conflict of Interest

The authors declare that the research was conducted in the absence of any commercial or financial relationships that could be construed as a potential conflict of interest.
