# Supplementary material for: Development and Validation of Nomograms Based on Gamma-Glutamyl Transpeptidase to Platelet Ratio for Hepatocellular Carcinoma Patients Reveal Novel Prognostic Value and the Ratio Is Negatively Correlated With P38MAPK Expression
Source: Front Oncol. 2020 Dec 3;10:548744. doi: 10.3389/fonc.2020.548744 (PMC7744698; doi:10.3389/fonc.2020.548744)
Supplement: Supplementary file 1 [file DataSheet_1.docx]

Supplementary Material

# Supplementary Figures and Tables

## Supplementary Figures


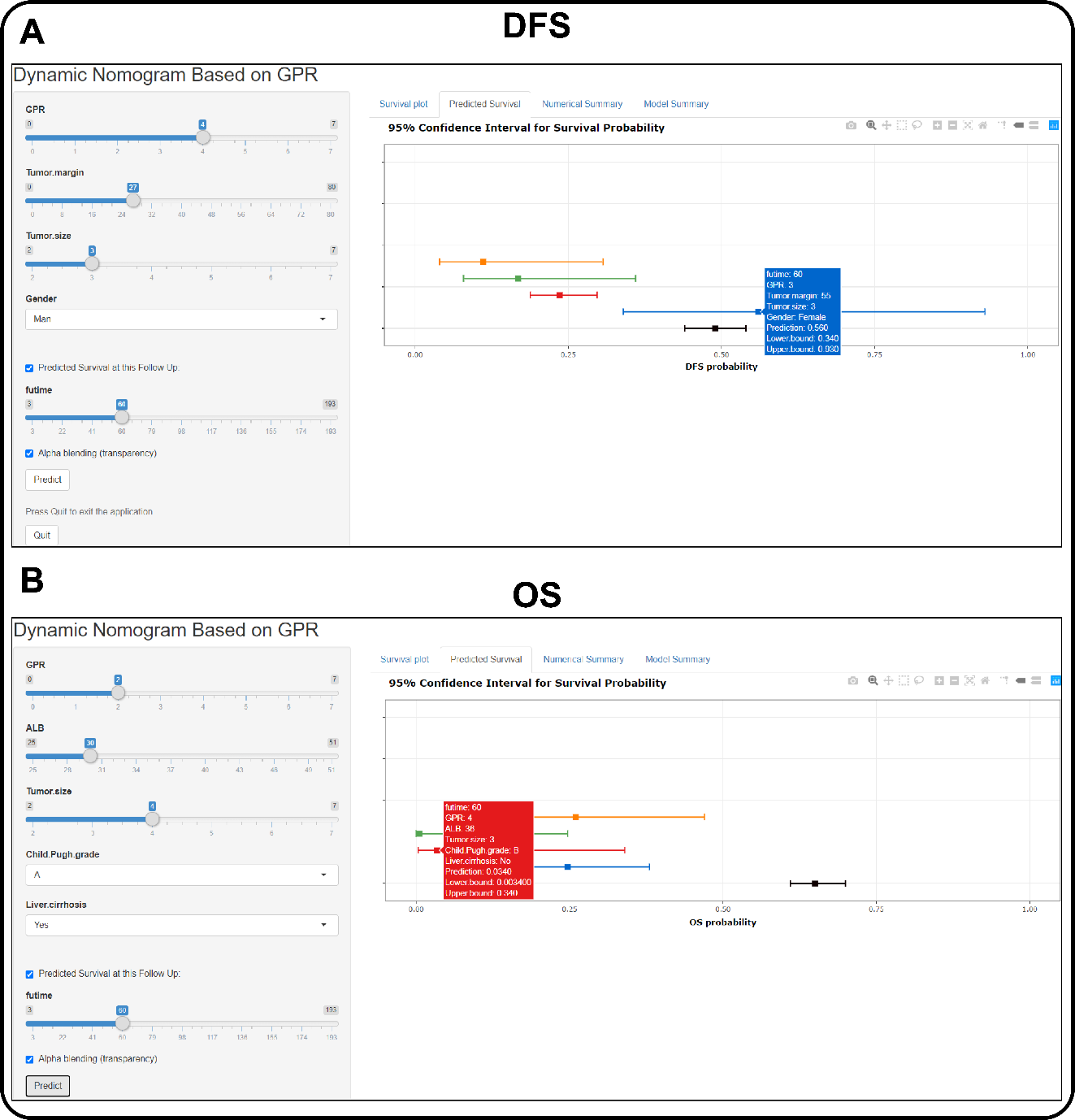


**Supplementary Figure 1.** **An Online version of our nomogram. Predicted the probability of DFS (****Supplementary appendix 1A) and OS (Supplementary appendix 1B) across time can be easily determined by inputting clinical features and reading output figures and tables generated by the webserver.**

**DFS:** <https://00logan00.shinyapps.io/Article_GPR_DFS/>

**OS:** <https://00logan00.shinyapps.io/Article_GPR_OS/>

## Supplementary Tables

**Supplementary Table 1. Association between the expression of GPR and p38MAPK**

| **P38MAPK** | **GPR** | |  | | | **Pearson’s contingency** | |  |
| --- | --- | --- | --- | --- | --- | --- | --- | --- |
|  | **High** | **Low** | |  | **Coefficient** | | **P-value** | |
| High | 23 | 33 | |  | 0.355 | | **0.001** | |
| Low | 22 | 5 | |  |  | |  | |

**Abbreviations**: GPR, gamma-glutamyl transpeptidase to platelet ratio.
